# Chiral 8-Amino-5,6,7,8-tetrahydroquinoline Derivatives in Metal Catalysts for the Asymmetric Transfer Hydrogenation of 1-Aryl Substituted-3,4-dihydroisoquinolines as Alkaloids Precursors

**DOI:** 10.3390/molecules28041907

**Published:** 2023-02-16

**Authors:** Giorgio Facchetti, Francesca Neva, Giulia Coffetti, Isabella Rimoldi

**Affiliations:** Dipartimento di Scienze Farmaceutiche, Università degli Studi di Milano, Via Venezian 21, 20133 Milano, Italy

**Keywords:** 1,2,3,4,5-pentamethylcyclopentadienyl metal complex, cyclic imines, diamine ligand, alkaloids precursors

## Abstract

Chiral diamines based on an 8-amino-5,6,7,8-tetrahydroquinoline backbone, known as CAMPY (**L1**), or the 2-methyl substituted analogue Me-CAMPY (**L2**) were employed as novel ligands in Cp* metal complexes for the ATH of a series of substituted dihydroisoquinolines (DHIQs), known for being key intermediates in the synthesis of biologically active alkaloids. Different metal-based complexes were evaluated in this kind of reaction, rhodium catalysts, **C3** and **C4**, proving most effective both in terms of reactivity and enantioselectivity. Although modest enantiomeric excess values were obtained (up to 69% *ee* in the case of substrate **I**), a satisfactory quantitative conversion was successfully fulfilled even in the case of the most demanding hindered substrates when La(OTf)_3_ was used as beneficial additive, opening up the possibility for a rational design of novel chiral catalysts alternatives to the Noyori-Ikariya (arene)Ru(II)/TsDPEN catalyst.

## 1. Introduction

Chiral amine fragment features in almost 40−45% of the small molecule pharmaceuticals and many other industrially relevant fine chemicals and agrochemicals, and it proves to be the key structural motif for their biological activity in more than 90% of top selling drugs [1,2]. Moreover, chiral amines are often used in the asymmetric synthetic protocols of structurally more complex molecules such as natural products as resolving agents or chiral auxiliaries. Thus, the increasing demand for enantiopure amines in the life sciences paved the way for the development of novel and sustainable synthetic routes for their efficient preparation [3]. Although the traditional resolution of diastereomeric salts is still used as a synthetic approach, the use of catalytic methods has been extremely widespread in recent decades. In particular, asymmetric catalysis based on transition metals provides the most straightforward method to access chiral amines. Indeed, many metal-catalyzed transformations for chiral amine synthesis have been reported so far with reductive amination, refs. [4,5,6] allylic amination, refs. [7,8] asymmetric N-H carbene insertion [9,10] or one-pot chemoenzymatic protocols [11,12,13]. Nonetheless, the asymmetric reduction of C=N bonds still provides one of the most accessible and practical strategies to afford enantiomerically enriched amines. ATH reaction (Asymmetric Transfer Hydrogenation) of imines is undoubtedly the most powerful approach within this field. ATH, indeed, offers excellent atom economy being followed by almost no or few byproducts. This represents a sustainable “green” process in the field of organometallic catalysis even to be preferred to the AH (Asymmetric Hydrogenation) analogue relying on the employment of a hydrogen source different to the hazardous gaseous hydrogen [14]. The most striking advances achieved in this field are surely due to the employment of the well-known Noyori–Ikariya catalysts of general formula [(arene)Ru(L)Cl] complexes, in which L represents *N*-tosyl-1,2-diphenylethylene-1,2- diamine (TsDPEN), which leads to exquisite enantioselectivity levels with any attempted modification to the tosylated diamine ligand. This results in less reactive or less selective catalysts. However, imines are more challenging substrates if compared to their oxygenated analogues ketones, due to easy hydrolysis, the presence of isomerism, and the possible poisoning and/or deactivation process involving the catalyst as a consequence of the strong coordination between the heteroatoms and the transition metal centers.

**Figure 1:**
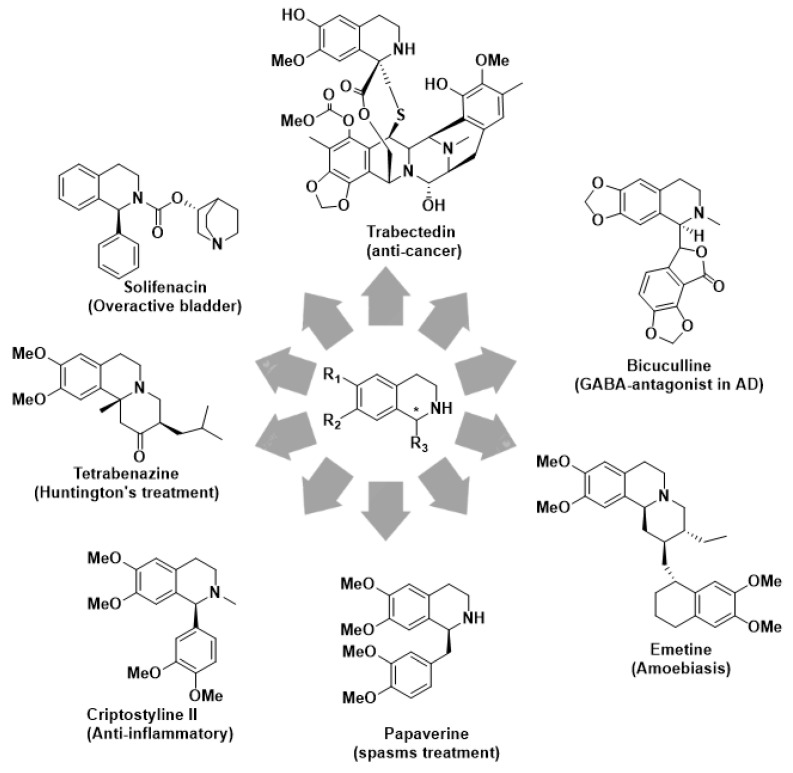
Tetrahydroisoquinoline core in biologically relevant natural and non-natural alkaloids.

Moreover, the aqueous media usually employed in the ATH protocol generally avoid the use of the most performing but moisture sensitive diphosphine ligands, refs. [15,16,17,18,19] thus evoking the need for new catalysts able to broaden the scope toward the most challenging dihydroisoquinolines (DHIQs). The related reduction products, namely tetrahydroisoquinolines (THIQs), indeed account for an important class of alkaloids and semi-synthetic derivatives endowed with multiple relevant biological properties (Figure 1) [20,21,22,23,24].

The THIQ core can be found in a tremendous variety of synthetic drugs as shown in Figure 1. Tetrabenazine, for instance, is the first drug approved for Huntington’s disease; Trabectedin is the first marine-derived anticancer drug approved for the treatment of soft tissue sarcomas; Solifenacin is indeed a muscarinic antagonist approved for the treatment of overactive bladder. More than 3000 compounds possess or derive from the THIQ substructures that have found application in the pharmaceutical industry. Most of them, unfortunately, are produced in inadequate amounts for being applied to drug development. Total synthesis is an alternative, but the complexity of certain structures and the stereochemistry control are still a challenge to face, encouraging for innovative synthetic approaches [25].

Starting from the wide expertise in the design and development of new transition metal-based catalysts achieved in our group and their application in the asymmetric hydrogenation along with our deep know-how in isoquinoline core synthesis and biological evaluation, refs. [26,27,28] this research paper deals with a series of transition metal catalysts bearing the chiral diamine ligand 8-amino-5,6,7,8-tetrahydroquinoline, known as CAMPY or the analogue 2-methyl-5,6,7,8-tetrahydroquinolin-8-amine, Me-CAMPY, [29] in association with a 1,2,3,4,5-pentamethylcyclopentadienyl (Cp*) moiety and their use in the ATH of a series of differently substituted 1-aryl-3,4-dihydroisoquinolines [30].

## 2. Results and Discussion

With the aim of developing a practical and sustainable process to access the series of valuable alkaloids precursors, we exploited two chiral 8-ammino-5,6,7,8-tetrahydroquinoline derivatives, (*R*)-CAMPY (**L1**) and (*R*)-Me-CAMPY (**L2**), as the source of chirality in transition metal complexes [30]. These diamine chelating ligands are different for both steric and electronic features influencing the reactivity of the corresponding metal complexes [30]. It’s worth noting that in this work we proved for the first time an optimized protocol for the synthesis of these two chiral diamines affording both the enantiomers of ligands **L1** and **L2** in excellent enantiopure form and with excellent product yield. The key synthetic step was the dynamic kinetic resolution of the starting material, the 5,6,7,8-tetrahydroquinolin-8-ol, carried out by lipase from *Candida antarctica*, overcoming the drawbacks occurring during the traditional resolution based on expensive chiral resolving agents often affording products in low yields and inadequate optical purity [31]. We deeply investigated the catalytic ability of the Cp* metal complexes of **L1** and **L2** in the asymmetric transfer hydrogenation (ATH) of a series of synthesized cyclic aromatic 1-aryl imines under mild aqueous reaction conditions [32]. Chiral **L1** and **L2** ligands featuring the pyridine backbone have already been demonstrated to perform extremely well in metal complexes when applied to homogeneous catalysis reactions, resulting in compatibility to both organic and aqueous media. Although the Noyori–Ikariya catalyst is highly effective and has been successfully applied in many synthetic protocols, the catalyst can be quite costly due to the high catalyst loadings that are often required (0.5–1.0 mol%) along with the ascertained Ts-DPEN instability under certain reaction conditions [33,34]. These drawbacks strongly encourage us to develop new chiral ligands for homogeneous catalysis with higher robustness in order to expand the scope of organic transformations by exploiting the pyridine core, beneficial in terms of chemical stability even in aqueous media and at different pH values [35,36,37]. Moreover, **L1** and **L2** could be easily synthesized starting from easily accessible materials in high enantiopure forms. For a preliminary screening study, 6,7-dimethoxy-1-phenyl-3,4-dihydroisoquinoline **I** was used as a model substrate in order to set up the reaction conditions (Table 1).

Some variables were taken into consideration, such as the use (or not) of the additive (CH_3_COOH, La(OTf)_3_ or Ag(OTf)_3_), which impacts the outcome of the reaction. The use of such additives stems from the well-documented hypothesis that imine reduction proceeds via iminium species [38,39,40]. Thus, the activation of the imine function via protonation or via Lewis acids may provide additional benefits in terms of conversion without impairing selectivity [41,42]. Other variables, tested for the optimization of the reaction conditions, were considered. These included the type and the amount of hydrogen donor (different ratio between HCOOH/TEA, the use of HCOOH or HCOONa) and the aqueous media employed (MeOH/H_2_O mixture, different buffer solution at different pH as CH_3_COOH 0.1 M at pH 5 or MOPS buffer 1.2 M at pH 7.8) (data not reported). An azeotropic mixture of formic acid and triethylamine (5:2) is definitely the most widely exploited hydrogen donor but it has been proven that the pH of the mixture has a strong influence on the enantioselectivity outcome of the reaction [27]. Under strong acidic conditions, e.g., at a high formic acid/triethylamine ratio, the primary amino function of the diamine ligand gets probably protonated, thus leading to its detachment from the metal center. This conformational change in the catalytic complex subsequent to the metal-diamine chelate may be responsible for a lower reduction rate along with a detrimental effect on the enantioselectivity of the catalyst [43,44]. Indeed, the use of a formic acid/triethylamine ratio adjusted to 1.1:1 ideally sets the reaction medium pH at a slightly acidic value of about 5.2. Under these favorable environmental features, the metal-based catalyst becomes more robust and efficient thus letting it establish those advantageous interactions with the protonated iminium ion that results in an enhancement of both the reaction rate and enantioselectivity. Regarding the solvent, the use of MeOH in water probably promotes the solubility of the substrate considering the chemical nature of the 1-aryl substituted-3,4-dihydroisoquinolines, generally less soluble in aqueous media.

**Figure 2:**
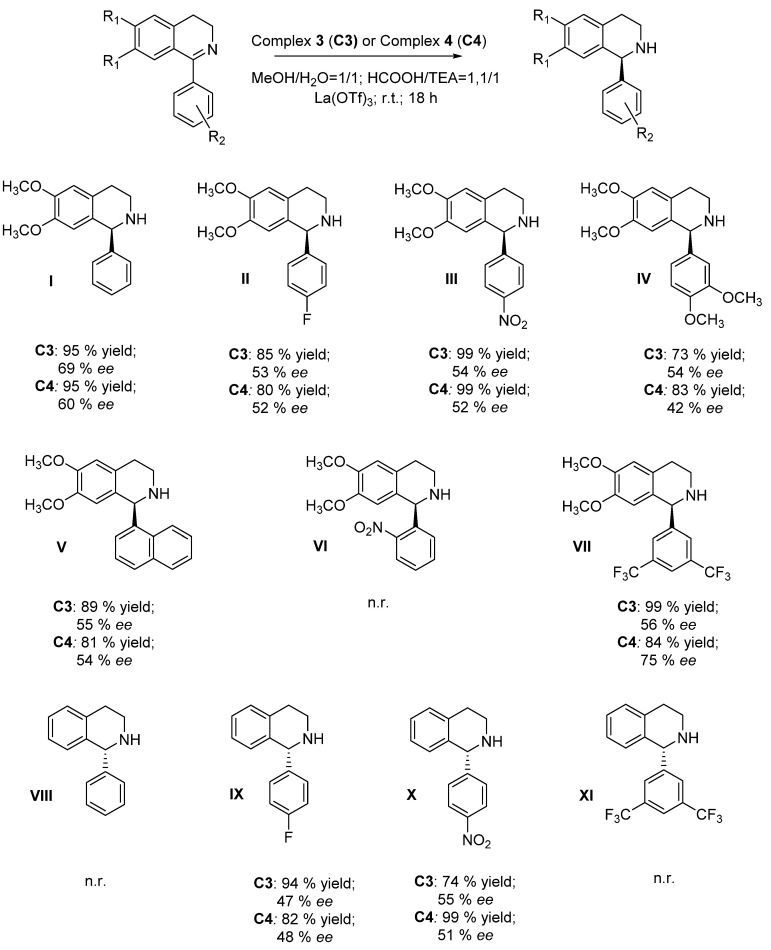
ATH of different 1-aryl imines using **C3** and **C4** as catalysts. n.r. = no reaction.

We applied the well-known Noyori–Ikariya catalyst RhCp*TsDPEN under the above-mentioned reaction conditions to the model substrate 6,7-dimethoxy-1-phenyl-3,4-dihydroisoquinoline, structurally related to the cryptostyline alkaloids [45]. The results confirmed the inadequacy of this catalyst in affording the asymmetric product in acceptable enantiopurity with only 7% *ee* (Table 1, line 1) [46]. Thus, considering our previous results obtained in the ATH reduction of salsolidine precursor, the 6,7-dimethoxy-1-methyl-3,4-dihydroisoquinoline with complex **C1** and **C2**, we have initially focused our attention on catalytic activity of these iridium complexes as expected to be the most promising in such type of reaction [27]. Contrary to our expectations in the case of **C1** and **C2,** the reduction of the selected model substrate proceeded with low enantioselectivity degree but the conversion rate increased when adding La(OTf)_3_ (Table 1, entries 2–5), confirming the Lewis acid activation effect exerted toward the substrate. When rhodium catalysts **C3** and **C4** were used, good results in terms of enantioselectivity were achieved for both the catalysts (69% *ee* for **C3** and 57% *ee* for **C4**). Quantitative conversions were realized only in the presence of La(OTf)_3_ as an additive. In the case of rhodium complexes the optimum aqueous medium relies on water in a mixture of 1:1 with methanol (Table 1, entries 6–9). In the case of the neutral ruthenium complexes **C5** and **C6**, the reaction didn’t proceed under any tested conditions. Indeed, only when La(OTf)_3_ was added was the reaction product obtained (although in trace amounts) with a sluggish enantiomeric excess in the presence of complex **C5** (Table 1, entry 11 vs. entries 10, 12 and 13). Thus, the obtained data underlined that the best results both in terms of enantioselectivity and reactivity were achieved by using rhodium Cp* complexes **C3** and **C4** in the presence of La(OTf)_3_ as additive. Finally, two achiral ligands **L3** (AMPY) and their tosylated analogue **L4** (Ts-AMPY) were also evaluated in the corresponding RhCp* complexes to shed light on both the structural flexibility of the ligand and the lack of an additional chirality, unless the chirality appeared at the metal center. The reaction proceeded in both cases, affording the product in a racemic mixture, thus unequivocally stressing the relevance of **L1** and **L2** chirality in the stereocontrol of the reaction (lines 14 and 15). The presence of the tosyl group in **C8**, however, proved essential for bringing about the conversion if compared to **C7**, analogously to the Ts-DPEN behavior [47]. After optimizing the reaction conditions for the ATH of the model substrate **I**, we decided to expand the scope. By the evaluation of the obtained preliminary results, we found the optimized protocol for the ATH reaction comprised a substrate:catalyst set at 100:1 ratio, in presence of 33% molar of the additive La(OTf)_3_, using HCOOH:TEA in ratio 1.1:1 at 30 °C for 18 h, in a water/methanol co-solvent system. Considering the possibility of evaluating the reactivity of the rhodium complexes, we set out to explore the structure-reactivity trend within a class of 1-aryl-3,4-dihydroisoquinolines, chosen for being biologically active alkaloids precursors (Figure 2).

**Figure 3:**
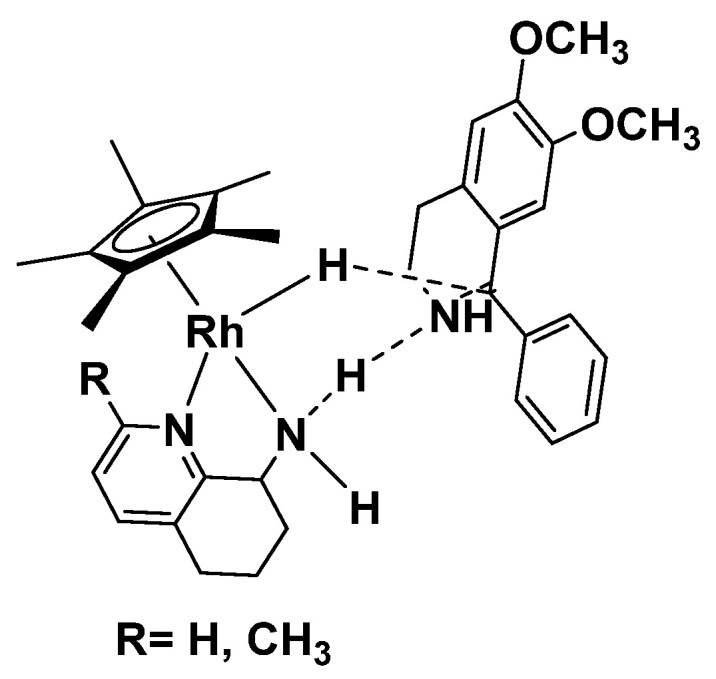
Hypothesis of interaction between the ligand **L1** or **L2** with the substrate.

As expected, for all the substrates bearing the 6,7-dimethoxy substituted 3,4-dihydroisoquinolines (substrates **I**–**VII**), the reaction proceeded with satisfying conversions with quantitative results for substrates **III** and **VII** by employing **C3** catalyst and **X** for the **C4** catalyzed reaction (see HPLC spectra in SI). The enantioselectivity, although a modest 69% *ee* for substrate **I**, proved reproducible, except for substrate **VI** for which the presence of a bulky nitrogroup in *ortho* position proved detrimental for the reduction of such a substrate that probably clashes with the rigid ligand **L1** and **L2**. Conversely, by depriving the substrates of the activating 6,7-dimethoxy substituents, as in the case of substrates **VIII-XI**, only the presence of an electron withdrawing group in *para* position allowed the reproducibility of the catalysts, affording the product good to quantitative yields (substrates **IX** and **X**). Probably, from the proposed mechanism of interaction between the enantioface of the ligand and the prochiral substrate (Figure 3), [48] it is reasonable to hypothesize that the effect exerted by the methyl group on **L2** results was irrelevant to stereo-differentiation between **C3** and **C4,** thus smoothing the enantiomeric excess for all the substrates.

## 3. Experimental

All manipulations involving air sensitive materials were carried out in an inert atmosphere glove box or using standard Schlenk line techniques, under an atmosphere of nitrogen or argon in oven-dried glassware. Reagents and solvents were purchased from Sigma-Aldrich and used without further purification. All tested compounds possessed a purity of >98% confirmed via elemental analyses (CHN) with a Perkin Elmer 2400 instrument (Waltham, MA, USA) and via NMR spectroscopy on a Bruker DRX Avance 600 MHz (Bruker, Germany). All the experiments were recorded at 298 K using TMS as internal standard. All the substrates and complexes were synthesized according to the procedure reported in the literature. HR-MS analyses were performed by using a QTof Synapt G2 Si spectrometer with an electrospray ionization source (Palmer, MA, USA). The MS spectra were obtained by direct infusion of a sample solution of 2 μg/mL in MeOH under ionization, ESI positive. Catalytic reactions were monitored by HPLC analysis using Merck-Hitachi L-7100 (Merck-Hitachi, Darmstadt, Germany) equipped with Detector UV6000LP and chiral column (Chiralcel OD-H, Chiralpak AD, Lux Cellulose-2 or Lux Amylose-2). Novozym 435 from Sigma-Aldrich (immobilized on acrylic resin, ≥5000 U/g, recombinant, expressed in *Aspergillus niger*). L3 was purchased from Sigma-Aldrich. L4 was purchased from ThermoFischer Scientific. Substrates **I**–**XI** were synthesized according to reported procedures and their spectra were in accordance to data reported in the literature [15]. HPLC spectra of products **I**–**XI** under optimized reaction conditions can be found in the Supplementary Materials, Figures S1–S9.

### 3.1. General Procedure for the Synthesis of **L1** and **L2**

The synthesis proceeded as reported in the literature [49].

A mixture of (±)-5,6,7,8-Tetrahydroquinoline-8-ol (1 eq.), vinyl acetate (5 eq.), lipase acrylic resin from *Candida antarctica* (0.5 eq.) and 4Å molecular sieves (5 eq.) in *i*-Pr_2_O (500 mL) were left under stirring for 30 h at 60 °C (25 mM final concentration). The reaction was monitored by HPLC equipped with chiral column Daicel AD-RH, (20% CH_3_CN in H_2_O; flow: 1.0 mL/min; retention time(*R*)-OAc: 10.8 min, (*S*)-OH: 13.5 min). Lipase and the molecular sieves were then removed by filtration on a celite pad. The filtrate was concentrated under vacuum and the two products, the (*S*)-5,6,7,8-tetrahydroquinoline-8-ol (88% yield) and the (*R*)-8-acetoxy-5,6,7,8-tetrahydroquinoline (86% yield), were separated by chromatography on silica gel with ethyl acetate and hexane in ratio 7:3. The (*R*)-8-acetoxy-5,6,7,8-tetrahydroquinoline enantiomer (1 eq.) and K_2_CO_3_ (4 eq.) in MeOH (10 mL) was stirred for 2 h at room temperature (160 mM final concentration). MeOH was removed under vacuum and the mixture treated with H_2_O and ethyl acetate. The organic phase was washed twice with brine (10 mL × 2) and dried on anhydrous Na_2_SO_4_. After evaporation of the solvent, the product was purified by column chromatography with ethyl acetate and hexane (1:1) to give (*R*)-OH- (88% yield). A solution of (*R*)-OH-or (S)-OH (1 eq.) in CH_2_Cl_2_ (10 mL), DMAP (6 eq.) MsCl (4 eq.) and NaN_3_ (50 eq.) was at 0 °C (60 mM final concentration). The mixture was kept under stirring at room temperature for 30 min, after which DMSO (10 mL) was added under stirring for an additional 6 h. The reaction was quenched with H_2_O and extracted with ethyl acetate and hexane in ratio 3:7. The organic phase was washed with H_2_O and brine, dried with Na_2_SO_4_ and concentrated. The product was purified by chromatography on silica gel using ethyl acetate and hexane in ratio 15:85 as eluent (89% yield). (*R*) or (*S*)-8-triazo-5,6,7,8-tetrahydroquinoline (1 eq.) and Pd-C (5% mol) in anhydrous EtOH (5 mL) was stirred for 3 h under an atmosphere of H_2_ (25 atm) at room temperature (140 mM final concentration). The Pd-C was removed by filtration on a celite pad and the ligand **L1** was obtained after concentration in vacuum as a pale-yellow oil. The same procedure was realized starting from 2-methyl-5,6,7,8-tetrahydroquinolin-8-amine for obtaining ligand **L2**.

(*R*)-**L1**: pale yellow oil (97% yield). ^1^H NMR (CDCl_3_, 300 MHz, 25 °C): *δ* = 1.68–1.75 (m, 2 H), 1.96 (m, 1 H), 2.24 (m, 1 H), 2.68–2.88 (m, 2 H), 3.45 (br, 2 H, NH_2_), 4.05 (t, *J* = 6.8 Hz, 1 H, H-8), 7.07 (dd, *J* = 7.3, 4.4 Hz, 1 H, H-3), 7.37 (d, *J* = 7.3 Hz, 1 H, H-4), 8.39 (d, *J* = 4.4 Hz, 1 H, H-2). ^13^C NMR (CDCl_3_, 75 MHz, 25 °C): *δ* = 19.8, 28.7, 31.3, 51.2, 121.8, 131.7, 136.8, 146.9, 158.3. MS (EI): *m*/*z* (%) 148 (M^+^, 100), 147 (77), 131 (21), 120 (78), 119 (60), 93 (36). MS (ESI) of C_9_H_12_N_2_ (*m*/*z*): calcd 148.1 (M^+^). found 148.1. [α]_D_^22^ = −51.0 (c = 0.55, CHCl_3_)

(*R*)-**L2**: pale yellow oil (82% yield). ^1^H NMR (CDCl_3_, 300 MHz, 25 °C): *δ* = 1.69–1.81 (m, 2H), 1.93–2.01 (m, 1H), 2.10–2.18 (m, 1H), 2.53 (s, 3H), 2.50–2.78 (m, 3H), 3.67 (t, *J* = 5.2 Hz, 1H), 7.05 (dd, *J* = 7.7, 4.7 Hz, 1H), 7.39 (d, *J* = 7.6 Hz, 1H), 8.40 (d, *J* = 4.6 Hz, 1H) ppm; ^13^C NMR (CDCl_3_, 75 MHz, 25 °C): *δ* = 19.55, 27.82, 28.85, 34.26, 59.56, 121.86, 132.46, 136.89, 146.86, 157,23 ppm. FTIR 3333.9, 3049.6, 2926.7, 2855.2, 2784.1, 1648.1, 1575.3, 1444.5, 1428.1, 1238.7, 1104.1, 782.2 cm^−1^. MS (ESI) of C_10_H_14_N_2_ (*m*/*z*): calcd 162.1, found 163.2 [M+1]^+^. [α]_D_^22^ = −20.8 (c 0.5, CH_2_Cl_2_).

### 3.2. General Procedure for ATH

The formic acid/triethylamine mixture 1.1:1 (50 equiv.) was charged into a sealed vial (2 mL), followed by the catalyst (1% mol) dissolved in a degassed water/methanol mixture 1:1 (1 mL). The resulting mixture was stirred for 30 min to activate the catalyst. The imine (16 mM final concentration) and La(OTf)_3_ (33% mol) was introduced at once and the mixture was vigorously stirred at 30 °C for 18 h. After the established reaction time, the sample was quenched by using a saturated solution of Na_2_CO_3_ (50 µL) and extracted with dichloromethane (3 × 200 µL). Combined extracts were dried over anhydrous sodium sulphate and the solvent was stripped off in a stream of nitrogen. The dry sample was dissolved in the selected eluent and directly analyzed via chiral HPLC according to the different analytical conditions reported for each substrate.

### 3.3. General Procedure for the Synthesis of Cp* Metal Complexes

The synthesis proceeded as reported in the literature [13].

In a 10 mL Schlenk tube, under nitrogen atmosphere, ligand (**L1** or **L2** or **L3** or **L4**) (1.1 equiv.) was dissolved in 3 mL of anhydrous ethanol. The proper dimeric pre-catalyst (0.5 equiv.) was added, and the suspension was heated at 50 °C for 3 h then warmed to room temperature and further stirred overnight (50 mM final concentration). The solvent was then evaporated under vacuum, and the obtained solid washed extensively with diethyl ether to finally afford the metal catalyst as a pure solid.

[Ir(Cp*)(R)-CAMPY(Cl)]Cl (**C1**): ^1^H NMR (300 MHz, CDCl_3_) *δ* 8.36 (d, *J* = 8.21 Hz, 1H), 7.59 (d, *J* = 8.69 Hz, 1H), 7.38–7.23 (m, 1H), 4.38–4.22 (m, 1H), 3.37–3.21 (m, 2H), 2.78–2.67 (m, 2H), 2.11–2.00 (m, 2H), 1.97 (15H) ppm. ^13^C NMR (75 MHz, CDCl_3_) *δ* 159.89, 148.87, 139.30, 136.22, 125.90, 87.54, 61.87, 31.83, 27.33, 21.42, 9.40 ppm. MS (ESI^+^) for C_19_H_27_ClN_2_Ir *m*/*z*: calculated 511.15, found 511.00 [M]^+^. Elemental analysis for C_19_H_27_Cl_2_N_2_Ir: calcd. C, 41.75; H, 4.98; N, 5.13; found C, 41.26; H, 4.47; N, 5.08.

[Ir(Cp*)(R)-Me-CAMPY (Cl)]Cl (**C2**): ^1^H NMR (300 MHz, CDCl_3_) *δ* 7.55 (d, *J* = 8.57 Hz, 1H), 7.28-7.21 (m, 1H), 4.23–4.09 (m, 1H), 3.57–3.42 (m, 2H), 2.96 (s, 3H), 2.82–2.66 (m, 2H), 2.16–1.98 (m, 2H), 1.88 (15H) ppm. ^13^C NMR (75 MHz, CDCl_3_) *δ* 159.12, 140.07, 139.51, 134.05, 126.31, 88.86, 87.94, 62.87, 35.78, 28.98, 26.91, 22.37, 9.81 ppm. MS (ESI^+^) for C_20_H_29_ClN_2_Ir *m*/*z*: calculated 525.16, found 525.19 [M]^+^. Elemental analysis for C_20_H_29_Cl_2_N_2_Ir: calcd. C, 42.85; H, 5.21; N, 5.00; found C, 43.26; H, 5.33; N, 5.06.

[RhCp*(R)-CAMPY (Cl)]Cl (**C3**): ^1^H NMR (300 MHz, CDCl_3_) *δ* 8.48 (d, *J* = 8.00 Hz, 1H), 7.57 (d, *J* = 8.10 Hz, 1H), 7.39–7.24 (m, 1H), 4.48–4.27 (m, 1H), 3.32–3.09 (m, 2H), 2.91–2.78 (m, 2H), 2.18–2.02 (m, 2H), 1.88 (s, 15H) ppm. ^13^C NMR (75 MHz, CDCl_3_) *δ* 159.23, 148.99, 139.62, 135.85, 125.77, 95.88, 95.78, 60.30, 32.11, 27.23, 21.19, 9.63 ppm. MS (ESI^+^) for C_19_H_27_ClN_2_Rh *m*/*z*: calculated 421.09, found 422.95 [M + H]^+^. Elemental analysis for C_19_H_27_Cl_2_N_2_Rh: calcd. C, 49.91; H, 5.95; N, 6.13; found C, 49.26; H, 6.01; N, 6.08.

[RhCp*(R)-Me-CAMPY (Cl)]Cl (**C4**): ^1^H NMR (300 MHz, CDCl_3_) *δ* 7.46 (d, *J* = 8.19 Hz, 1H), 7.24 (d, *J* = 6.41 Hz, 1H), 4.59–4.36 (m, 1H), 3.58–3.48 (m, 2H), 2.96 (s, 3H), 2.89–2.71 (m, 2H), 2.16–1.94 (m, 2H), 1.89 (s, 15H) ppm. ^13^C NMR (75 MHz, CDCl_3_) *δ* 159.77, 157.92, 139.34, 131.86, 125.11, 106.66, 105.33, 102.02, 99.13, 95.90, 58.41, 33.28, 28.01, 21.44, 19.55, 9.83 ppm. MS (ESI^+^) for C_20_H_29_ClN_2_Rh *m*/*z*: calculated 435.11, found 436.05 [M + H]^+^. Elemental analysis for C_20_H_29_Cl_2_N_2_Rh: calcd. C, 50.97; H, 6.20; N, 5.94; found C, 50.98; H, 6.11; N, 5.88.

RuCp*(R)-CAMPY (Cl) (**C5**): ^1^H NMR (300 MHz, CDCl_3_) δ 8.34 (d, *J* = 7.89 Hz, 1H), 7.62 (d, *J* = 8.21 Hz, 1H), 7.39–7.28 (m, 1H), 4.45–4.14 (m, 1H), 3.33–3.18 (m, 2H), 2.91–2.63 (m, 2H), 2.19–2.00 (m, 2H), 1.91 (s, 15H) ppm. ^13^C NMR (75 MHz, CDCl_3_) *δ* 159.67, 157.86, 133.31, 132.01, 125.45, 107.00, 105.27, 101.97, 99.15, 96.12, 58.21, 33.38, 28.13, 21.33, 10.11 ppm. MS (ESI^+^) for C_19_H_27_ClN_2_Ru *m*/*z*: calculated 420.09, found 421.11 [M + H]^+^. Elemental analysis for C_19_H_27_ClN_2_Ru: calcd. C, 54.34; H, 6.48; N, 6.67; found C, 53.78; H, 6.13; N, 6.56.

RuCp*(R)-Me-CAMPY(Cl) (**C6**): ^1^H NMR (300 MHz, CDCl_3_) *δ* 7.66 (d, *J* = 7.12 Hz, 1H), 7.48–7.39 (m, 1H), 4.44–4.19 (m, 1H),3.31–3.18 (m, 2H), 3.11 (s, 3H), 2.87–2.64 (m, 2H), 2.18–1.98 (m, 2H), 1.95 (s, 15H) ppm. ^13^C NMR (75 MHz, CDCl_3_) *δ* 159.61, 158.52, 139.28, 132.46, 125.01, 105.21, 104.72, 101.96, 99.17, 96.00, 58.33, 33.36, 27.58, 21.32, 18.46, 10.31 ppm. MS (ESI^+^) for C_20_H_29_ClN_2_Ru *m*/*z*: calculated 434.11, found 435.12 [M + H]^+^. Elemental analysis for C_20_H_29_ClN_2_Ru: calcd. C, 55.35; H, 6.74; N, 6.46; found C, 53.78; H, 6.13; N, 6.56.

[RhCp*-AMPY (Cl)]Cl (**C7**): ^1^H NMR (300 MHz, CDCl_3_) *δ* 8.58 (d, *J* = 7.63 Hz, 1H), 7.96–7.70 (m, 1H), 7.52–7.34 (m, 2H), 4.44 (dd, *J* = 8.23, 4.81 Hz, 2H), 1.89 (s, 15H) ppm. ^13^C NMR (75 MHz, CDCl_3_) *δ* 161.99, 150.68, 139.25, 125.51, 122.16, 95.88, 95.77, 51.68, 9.50 ppm. MS (ESI^+^) for C_16_H_23_ClN_2_Rh *m*/*z*: calculated 381.06, found 404.04 [M + Na]^+^. Elemental analysis for C_20_H_29_Cl_2_N_2_Rh: calcd. C, 46.07; H, 5.56; N, 6.72; found C, 46.44; H, 6.01; N, 6.78.

RhCp*-Ts-AMPY(Cl) (**C8**): ^1^H NMR (300 MHz, CDCl_3_) *δ* 9.19 (d, *J* = 5.64 Hz, 1H), 7.81 (t, *J* = 6.2 Hz, 1H), 7.65 (d, *J* = 8.01 Hz, 2H), 7.35 (d, *J* = 7.63 Hz, 2H), 7.15 (d, *J* = 7.89 Hz, 2H), 4.03 (q, *J* = 17.12 Hz, 2H), 2.46 (s, 15H), 2.28 (s, 3H) ppm. ^13^C NMR (75 MHz, CDCl_3_) *δ* 162.04, 151.12, 142.32, 139.89, 139.02, 129.64, 127.83, 126.77, 121.85, 96.32, 95.53, 50.87, 21.44, 9.76 ppm. MS (ESI^+^) for C_23_H_28_ClN_2_O_2_RhS *m*/*z*: calculated 534.06, found 535.07 [M + H]^+^. Elemental analysis for C_23_H_28_ClN_2_O_2_RhS: calcd. C, 51.65; H, 5.28; N, 5.24; found C, 51.37; H, 5.17; N, 5.23.

### 3.4. Analytical Conditions

The products were analyzed by ^1^H NMR to determinate the molar conversion whereas the enantiomeric excess was evaluated by HPLC analysis and the absolute configuration was assigned by comparison with literature references [15].

*6,7-dimethoxy-1-phenyl-1,2,3,4-tetrahydroisoquinoline* (**I**): *R*-isomer: 11.1 min (min); *S*-isomer: 15.4 min (maj); column: Chiralcel OD-H, eluent: 2-propanol/hexane = 30/70 (0.01% DEA), flow = 0.7 mL/min, λ = 285 nm.

*1-(4-fluorophenyl)-6,7-dimethoxy-1,2,3,4-tetrahydroisoquinoline* (**II**): *R*-isomer: 21.0 min (min); *S*-isomer: 23.9 min (maj); column: Chiralpak AD-H, eluent: 2-propanol/hexane = 10/90, flow = 0.8 mL/min, λ = 220 nm.

*6,7-dimethoxy-1-(4-nitrophenyl)-1,2,3,4-tetrahydroisoquinoline* (**III**): *R*-isomer: 27.9 min (min); *S*-isomer: 35.7 min (maj); column: Chiralcel OD-H, eluent: 2-propanol/hexane = 30/70 (0.01% DEA), flow = 0.7 mL/min, λ = 285 nm.

*1-(3,4-dimethoxyphenyl)-6,7-dimethoxy-1,2,3,4-tetrahydroisoquinoline* (**IV**): *R*-isomer: 23.7 min (min); *S*-isomer: 35.0 min (maj); column: Chiralcel OD-H, eluent: 2-propanol/hexane = 30/70 (0.01% DEA), flow = 0.7 mL/min, λ = 285 nm.

*6,7-dimethoxy-1-(naphthalen-1-yl)-1,2,3,4-tetrahydroisoquinoline* (**V**): 1° isomer: 14.7 min (min); 2° isomer: 16.4 min (maj); column: Chiralcel OD-H, eluent: 2-propanol/hexane = 30/70 (0.01% DEA), flow = 0.7 mL/min, λ = 285 nm.

*6,7-dimethoxy-1-(2-nitrophenyl)-1,2,3,4-tetrahydroisoquinoline* (**VI**): 1° isomer: 15.2 min (min); 2° isomer: 17.3 min (maj) column: Chiralcel OD-H, eluent: 2-propanol/hexane = 30/70 (0.01% DEA), flow = 0.7 mL/min, λ = 285 nm.

*1-(3,5-bis(trifluoromethyl)phenyl)-6,7-dimethoxy-1,2,3,4-tetrahydroisoquinoline* (**VII**): 1° isomer: 6.9 min (maj); 2° isomer: 9.4 min (min); column: Lux Amilose-2, eluent: 2-propanol/hexane = 10/90 (0.01% DEA), flow = 0.8 mL/min, λ = 220 nm.

*1-phenyl-1,2,3,4-tetrahydroisoquinoline* (**VIII**): *S*-isomer: 13.1 (min); *R*-isomer: 15.4 (maj); column: Chiralpak AD, eluent: 2-propanol/hexane = 4/96, flow = 0.8 mL/min, λ = 240 nm.

*1-(4-fluorophenyl)-1,2,3,4-tetrahydroisoquinoline* (**IX**): *S*-isomer: 7.0 (min); *R*-isomer: 7.5 (maj); column: AD-H, eluent: 2-propanol/hexane = 10/90, flow = 1.0 mL/min, λ = 220 nm.

*1-(4-nitrophenyl)-1,2,3,4-tetrahydroisoquinoline* (**X**): *S*-isomer: 17.9 (min); *R*-isomer: 19.9 (maj); column: Lux Amylose-2, eluent: 2-propanol/hexane = 10/90 (0.01% DEA), flow = 0.8 mL/min, λ = 254 nm.

*1-(3,5-bis(trifluoromethyl)phenyl)-1,2,3,4-tetrahydroisoquinoline* (**XI**): 1° isomer: 4.4 (min); 2° isomer: 5.0 (maj); column: Lux Cellulose-2, eluent: 2-propanol/hexane = 5/110, flow = 0.6 mL/min, λ = 220 nm.

## 4. Conclusions

In conclusion, in this paper, we described the search for novel metal catalysts endowed with a broader compatibility reaction toward the most challenging substrates such as the asymmetric transfer hydrogenation of 1-aryl substituted-3,4-dihydroisoquinolines, known for being the skeleton of biologically active molecules as alkaloids. Indeed, we set up novel rhodium catalysts based on the use of the two diamines **L1** and **L2** as chiral ligands, (*R*)-CAMPY and (*R*)-Me-CAMPY, comparing their reactivity with the achiral **L3** (AMPY) and **L4** (Ts-AMPY). The chirality of L1 and L2 proved essential to the stereocontrol of the reaction. Although a modest enantiomeric excess was achieved (<70% *ee*), probably arising from an unfavorable matching between the rigidity of the ligand skeleton and the steric hindrance of the substrates, these results are evinced for both the complex **C3** and **C4,** affording the product in quantitative yields even in the case of sterically hindered substrates (**III**, **VII** and **X**). The rhodium complexes outperforming the iridium and ruthenium analogues. The ineffective role played by the methyl group introduced in **C4** unfortunately resulted in an averaged outcome in terms of enantioselection, thus suppressing its additive effect arising from increasing steric hindrance and modifying electronic properties of the chiral ligand **L2**. Conversely, the important role played by the Lewis acidic additive, in particular La(OTf)_3_, was highlighted, whose activation effect toward the substrate was confirmed by the accelerating impact on the ATH conversion even in the case of the more challenging substituted DHIQs.

## Figures and Tables

**Table 1:** Screening of different 1,2,3,4,5-pentamethylcyclopentadiene metal complexes in ATH of substrate **I**.

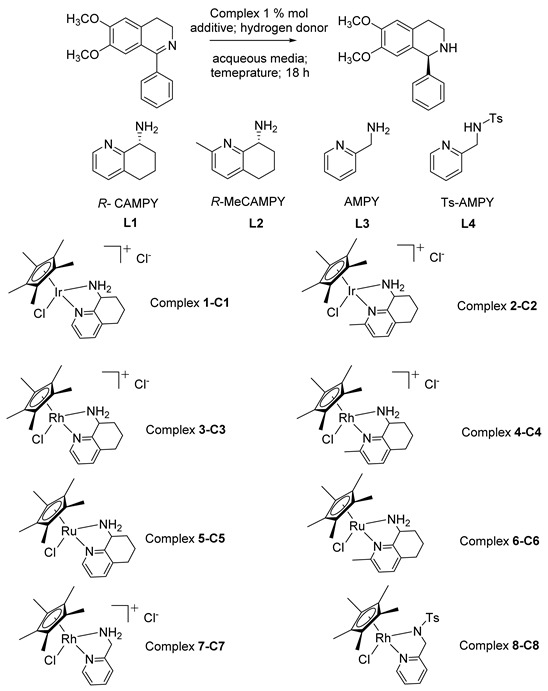						
**Entry**	**Complex**	**Additive**	**Hydrogen Donor**	**Media**	**Conversion %**	***ee* %**
**1**	RhCp*TsDPEN	none	HCOOH/TEA 1.1:1	H_2_O/MeOH 1:1	84	7
2	**C1**	none	HCOOH/TEA 1.1:1	MOPS buffer 1.2 M pH 7.8	94	27
3	**C1**	La(OTf)_3_	HCOOH	H_2_O/MeOH 1:1	99	45
4	**C2**	none	HCOOH/TEA 1.1:1	K_2_HPO_4_/NaH_2_PO_4_ 0.1 M pH 8	38	22
5	**C2**	La(OTf)_3_	HCOOH	MOPS buffer 1.2 M pH 7.8	95	18
6	**C3**	none	HCOOH/TEA 1.1:1	H_2_O/MeOH 1:1	49	69
7	**C3**	La(OTf)_3_	HCOOH/TEA 1.1:1	H_2_O/MeOH 1:1	95	69
8	**C4**	none	HCOOH/TEA 1.1:1	H_2_O/MeOH 1:1	60	57
9	**C4**	La(OTf)_3_	HCOOH/TEA 1.1:1	H_2_O/MeOH 1:1	95	60
10	**C5**	none	n.r.	n.r.		
11	**C5**	La(OTf)_3_	HCOOH/TEA 1.1:1	H_2_O/MeOH 1:1	>10	5
12	**C6**	none	n.r.	n.r.		
13	**C6**	La(OTf)_3_	n.r.	n.r.		
**14**	**C7**	none	HCOOH/TEA 1.1:1	H_2_O/MeOH 1:1	13	rac
**15**	**C8**	none	HCOOH/TEA 1.1:1	H_2_O/MeOH 1:1	94	rac

All reactions were carried out for 18 h at 30 °C using 1 mol % metal complex in the selected water medium with 50 equiv. of hydrogen donor (formic acid/triethylamine 1.1/1), [sub]f = 16 mM. Conversion and enantiomeric excess were determined using NMR spectroscopy and HPLC equipped with chiral column. n.r.= no reaction.

## Data Availability

Not applicable.

## Supplementary Materials

The following are available online at https://www.mdpi.com/article/10.3390/molecules28041907/s1, HPLC spectra of products **I**–**XI** under optimized reaction conditions. Figure S1: Spectra of substrate **I** with **C3** (69 % *ee*) and **C4** (60 % *ee*) respectively; Figure S2: Spectra of substrate II with **C3** (53 % *ee*) and **C4** (52 % *ee*) respectively; Figure S3: Spectra of substrate **III** with **C3** (54 % *ee*) and **C4** (52 % *ee*) respectively; Figure S4: Spectra of substrate **IV** with **C3** (54 % *ee*) and **C4** (42 % *ee*) respectively; Figure S5: Spectra of substrate **V** with **C3** (55 % *ee*) and **C4** (54 % *ee*) respectively; Figure S6: Spectra of substrate **VII** with **C3** (56 % *ee*) and **C4** (75 % *ee*) respectively; Figure S7.:Spectra of substrate **IX** with **C3** (47 % *ee*) and **C4** (48 % *ee*) respectively; Figure S8: Spectra of substrate **X** with **C3** (55 % *ee*) and **C4** (51 % *ee*) respectively; Figure S9: Spectra of substrate **I** with **C8**.

## Sample Availability

Samples of the compounds **I**–**XI** are available from the authors.
