# Supplementary material for: Chiral 8-Amino-5,6,7,8-tetrahydroquinoline Derivatives in Metal Catalysts for the Asymmetric Transfer Hydrogenation of 1-Aryl Substituted-3,4-dihydroisoquinolines as Alkaloids Precursors
Source: Molecules. 2023 Feb 16;28(4):1907. doi: 10.3390/molecules28041907 (PMC9962878; doi:10.3390/molecules28041907)
Supplement: Supplementary file 1 [file molecules-28-01907-s001.zip › molecules-2196719-supplementary.pdf]

SUPPLEMENTARY MATERIALS

Chiral 8-Amino-5,6,7,8-tetrahydroquinoline Derivatives in Metal Catalysts for the Asymmetric Transfer Hydrogenation of 1-Aryl Substituted-3,4-dihydroisoquinolines as Alkaloids Precursors

Giorgio Facchetti *, Francesca Neva, Giulia Coffetti and Isabella Rimoldi *

Dipartimento di Scienze Farmaceutiche, Università degli Studi di Milano, Via Venezian 21, 20133 Milano, Italy
* Correspondence: giorgio.facchetti@unimi.it (G.F.); isabella.rimoldi@unimi.it (I.R.)

HPLC SPECTRA OF ATH PRODUCTS I-XI UNDER OPTIMIZED REACTION CONDITIONS

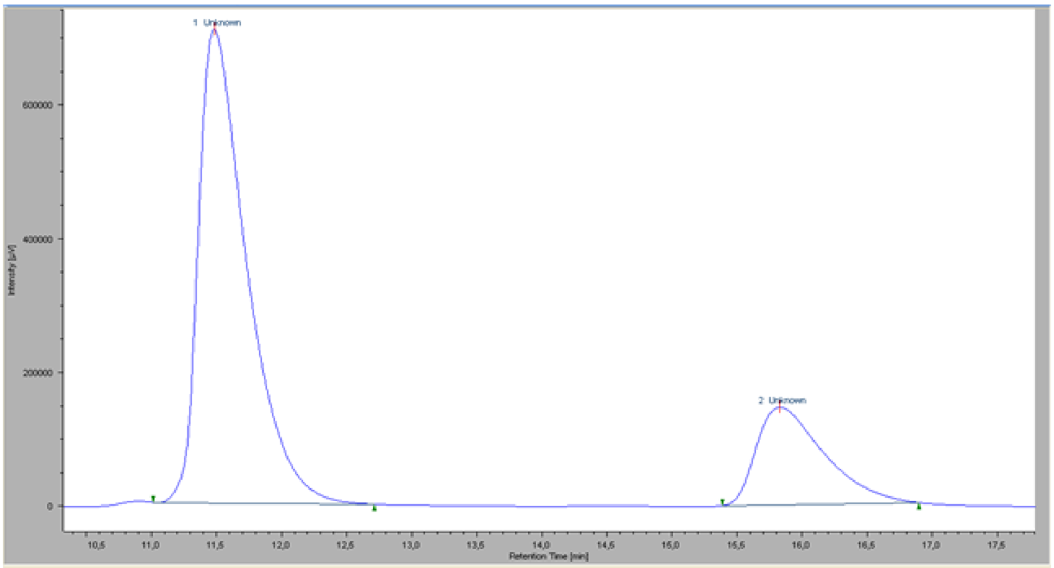

Peak Info. Table

#	Peak Name	tR	Area	Height	Area%	Height%	Peak Start	Peak End	Base Start	Base End	Peak Mark
1	Unknown	11,317	4347953	194037	84,127	88,904	10,826	12,008	10,826	12,008	Manual
2	Unknown	15,783	820391	24217	15,873	11,096	15,367	16,676	15,367	16,676	Manual

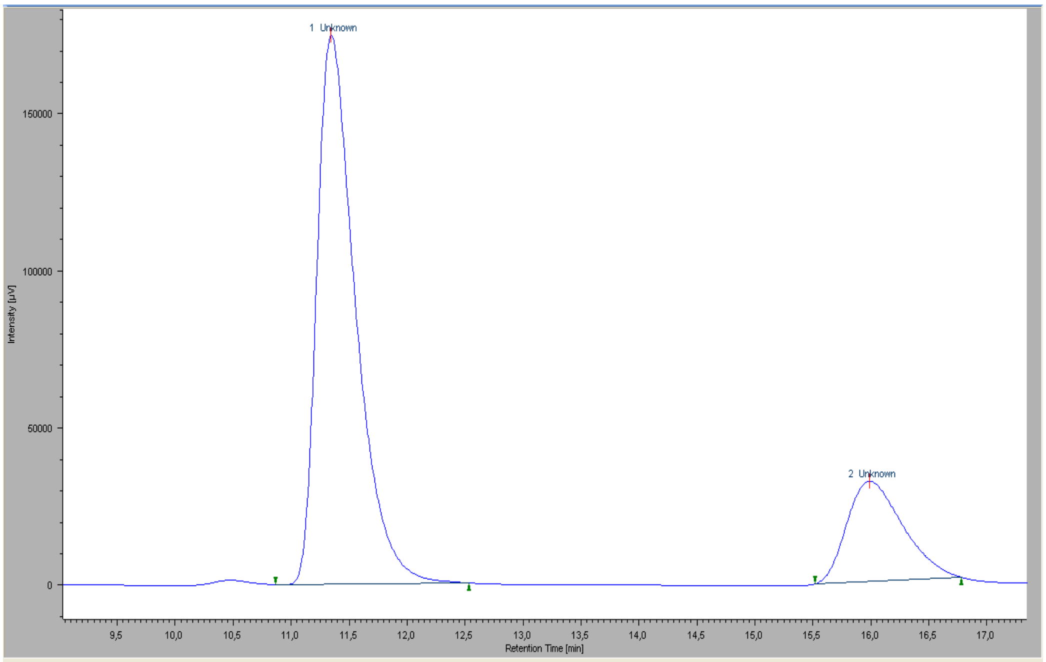

Peak Info. Table

#	Peak Name	tR	Area	Height	Area%	Height%	Peak Start	Peak End	Base Start	Base End	Peak Mark
1	Unknown	11,342	4044894	174350	79,178	84,581	10,867	12,533	10,867	12,533	Manual
2	Unknown	15,983	1063703	31784	20,822	15,419	15,517	16,775	15,517	16,775	Manual

Figure S1. Spectra of substrate **I** with **C3** (69 % e.e.) and **C4** (60 % e.e.) respectively.

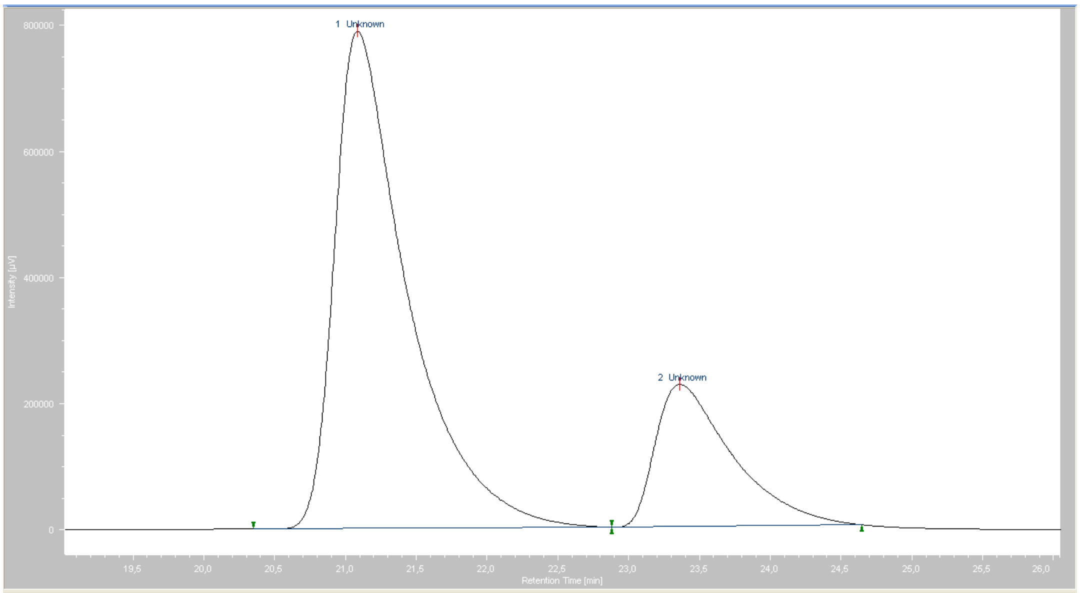

Peak Info. Table

#	Peak Name	tR	Area	Height	Area%	Height%	Peak Start	Peak End	Base Start	Base End	Peak Mark
1	Unknown	21,083	28301465	787953	76,928	77,769	20,350	22,875	20,350	22,875	Manual
2	Unknown	23,358	8488221	225240	23,072	22,231	22,875	24,642	22,875	24,642	Manual

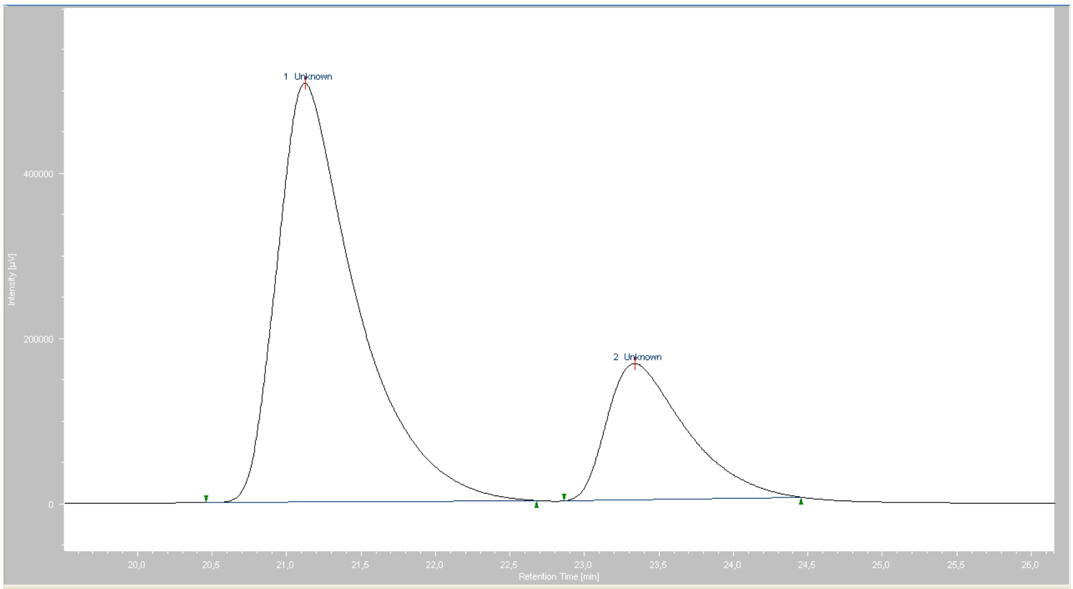

Peak Info. Table

#	Peak Name	tR	Area	Height	Area%	Height%	Peak Start	Peak End	Base Start	Base End	Peak Mark
1	Unknown	21,125	18845109	505619	75,609	75,460	20,458	22,675	20,458	22,675	Manual
2	Unknown	23,333	6079301	164427	24,391	24,540	22,858	24,450	22,858	24,450	Manual

Figure S2. Spectra of substrate **II** with **C3** (53 % e.e.) and **C4** (52 % e.e.) respectively.

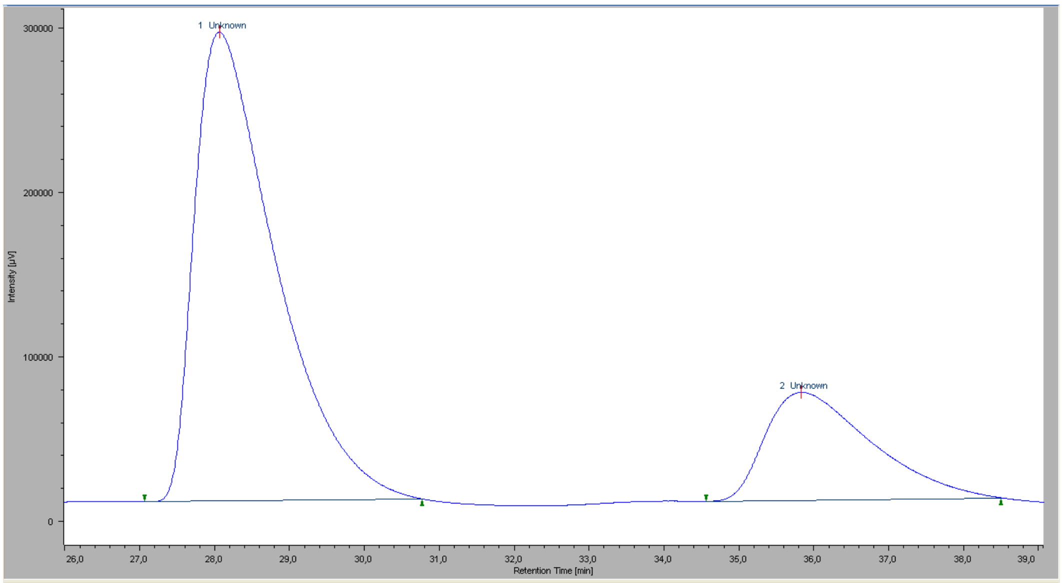

Peak Info. Table

#	Peak Name	tR	Area	Height	Area%	Height%	Peak Start	Peak End	Base Start	Base End	Peak Mark
1	Unknown	28,067	21427773	284550	76,928	81,291	27,067	30,767	27,067	30,767	Manual
2	Unknown	35,825	6426374	65490	23,072	18,709	34,558	38,492	34,558	38,492	Manual

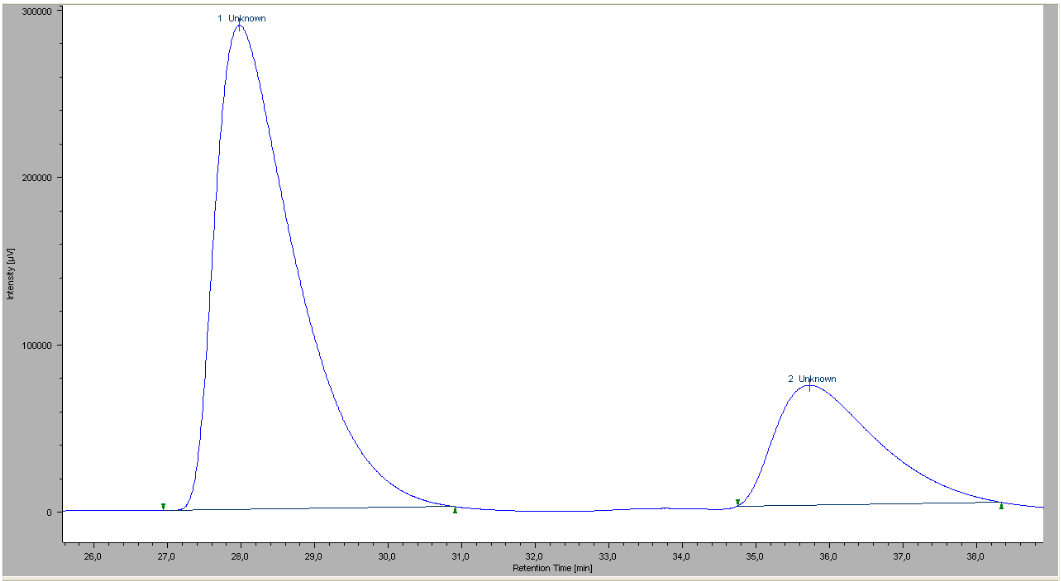

Peak Info. Table

#	Peak Name	tR	Area	Height	Area%	Height%	Peak Start	Peak End	Base Start	Base End	Peak Mark
1	Unknown	27,975	22044520	288751	76,241	80,163	26,942	30,900	26,942	30,900	Manual
2	Unknown	35,725	6869839	71455	23,759	19,837	34,742	38,325	34,742	38,325	Manual

Figure S3. Spectra of substrate II**I** with **C3** (54 % e.e.) and **C4** (52 % e.e.) respectively.

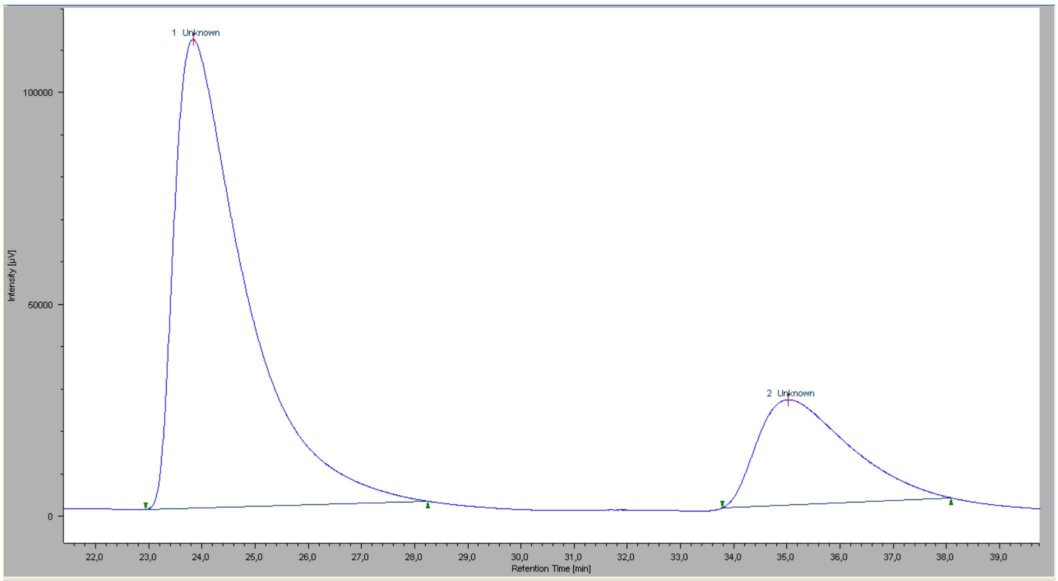

Peak Info. Table

#	Peak Name	tR	Area	Height	Area%	Height%	Peak Start	Peak End	Base Start	Base End	Peak Mark
1	Unknown	23,833	10233838	110377	77,497	81,651	22,950	28,250	22,950	28,250	Manual
2	Unknown	35,017	2971657	24805	22,503	18,349	33,775	38,067	33,775	38,067	Manual

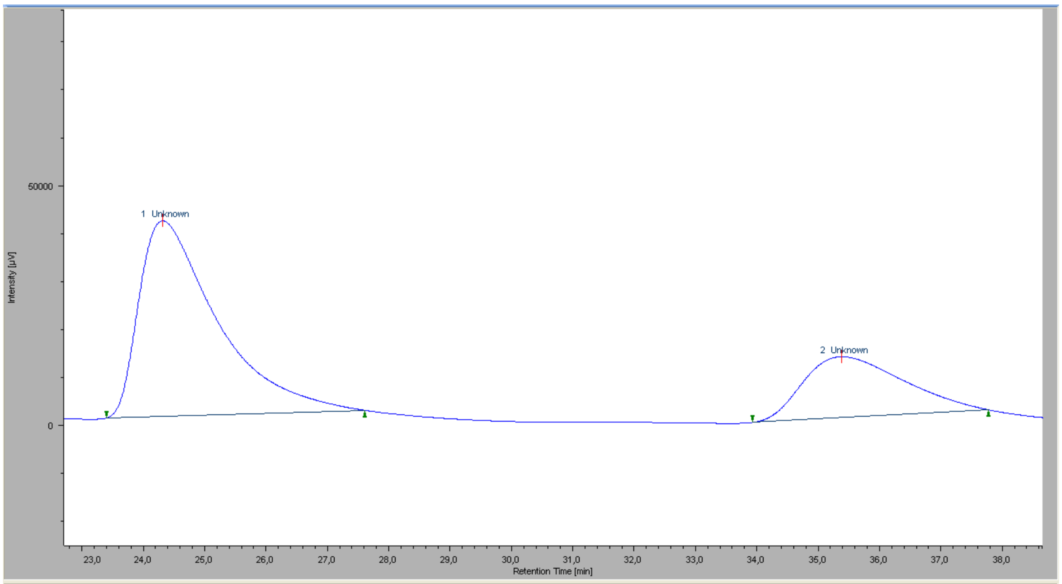

Peak Info. Table

#	Peak Name	tR	Area	Height	Area%	Height%	Peak Start	Peak End	Base Start	Base End	Peak Mark
1	Unknown	24,317	3601950	40700	71,504	76,213	23,400	27,600	23,400	27,600	Manual
2	Unknown	35,375	1435479	12703	28,496	23,787	33,925	37,767	33,925	37,767	Manual

Figure S4. Spectra of substrate **IV** with **C3** (54 % e.e.) and **C4** (42 % e.e.) respectively.

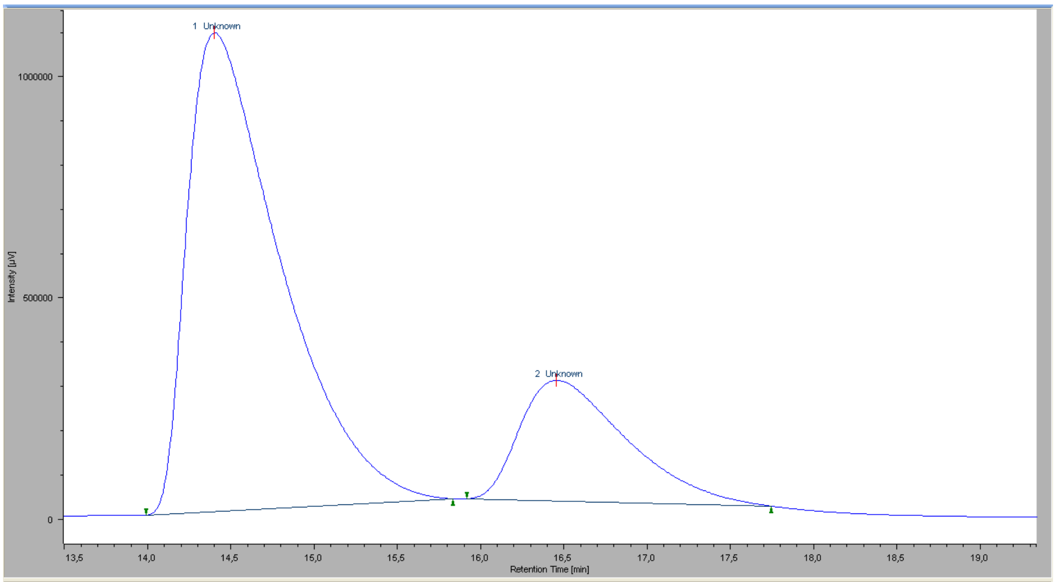

Peak Info. Table

#	Peak Name	tR	Area	Height	Area%	Height%	Peak Start	Peak End	Base Start	Base End	Peak Mark
1	Unknown	14,400	42214511	1080622	77,712	79,875	13,992	15,833	13,992	15,833	Manual
2	Unknown	16,450	12107480	272269	22,288	20,125	15,917	17,742	15,917	17,742	Manual

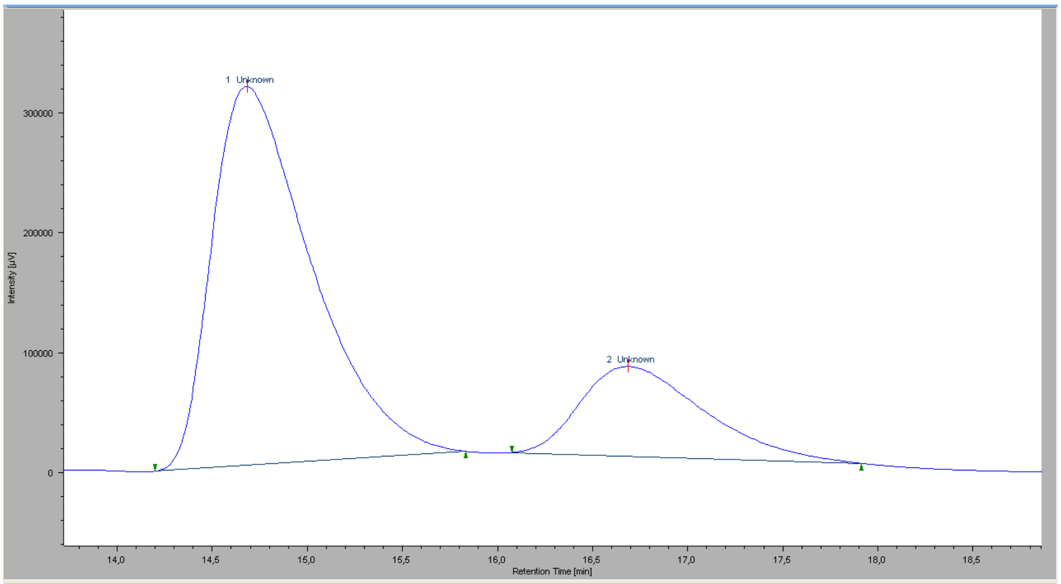

Peak Info. Table

#	Peak Name	tR	Area	Height	Area%	Height%	Peak Start	Peak End	Base Start	Base End	Peak Mark
1	Unknown	14,683	11477633	315250	77,331	80,869	14,200	15,833	14,200	15,833	Manual
2	Unknown	16,683	3364660	74576	22,669	19,131	16,075	17,908	16,075	17,908	Manual

Figure S5. Spectra of substrate **V** with **C3** (55 % e.e.) and **C4** (54 % e.e.) respectively.

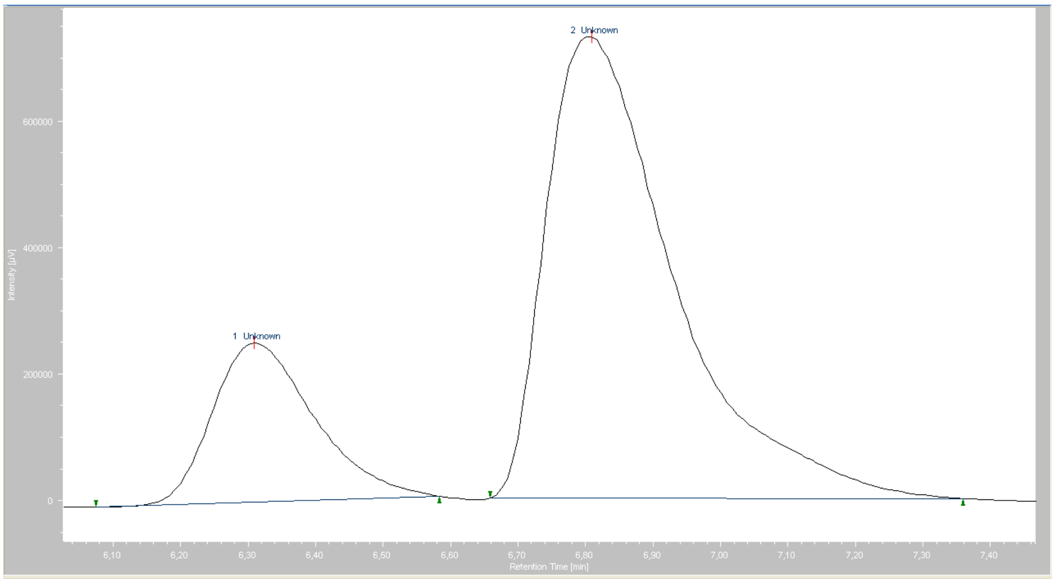

Peak Info. Table

#	Peak Name	tR	Area	Height	Area%	Height%	Peak Start	Peak End	Base Start	Base End	Peak Mark
1	Unknown	6,308	2618190	251287	21,475	25,671	6,075	6,583	6,075	6,583	Manual
2	Unknown	6,808	9573823	727591	78,525	74,329	6,658	7,358	6,658	7,358	Manual

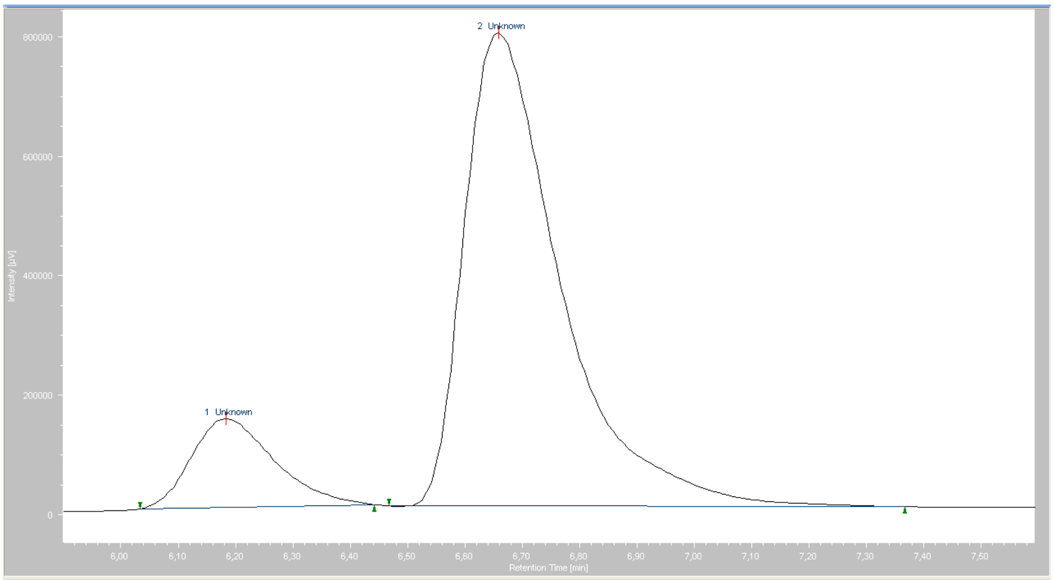

Peak Info. Table

#	Peak Name	tR	Area	Height	Area%	Height%	Peak Start	Peak End	Base Start	Base End	Peak Mark
1	Unknown	6,183	1472478	147668	13,766	15,730	6,033	6,442	6,033	6,442	Manual
2	Unknown	6,658	9224294	791118	86,234	84,270	6,467	7,367	6,467	7,367	Manual

Figure S6. Spectra of substrate **VII** with **C3** (56 % e.e.) and **C4** (75 % e.e.) respectively.

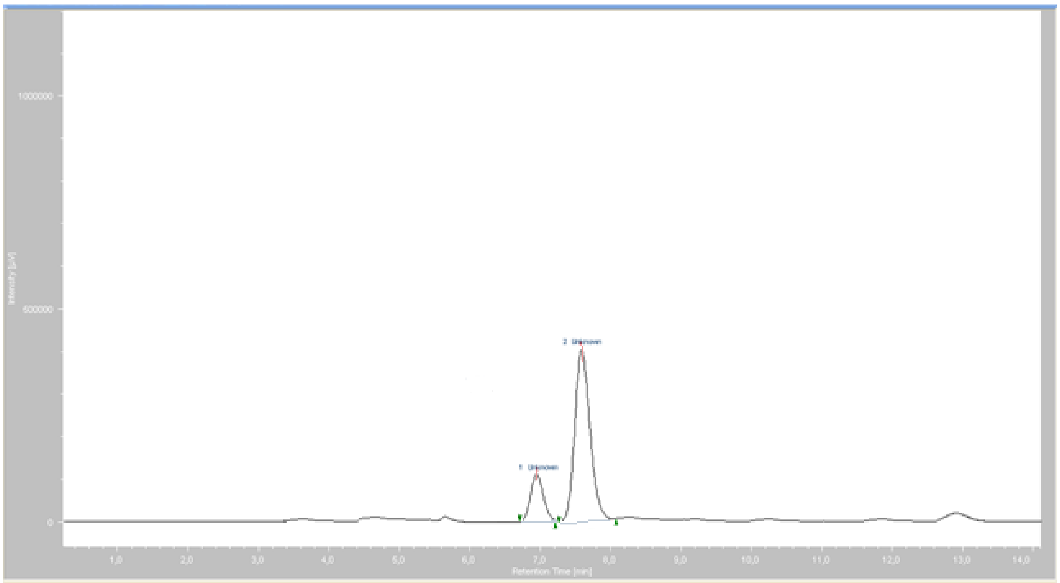

Peak Info. Table

#	Peak Name	tR	Area	Height	Area%	Height%	Peak Start	Peak End	Base Start	Base End	Peak Mark
1	Unknown	7,026	7184272	269821	73,882	80,419	11,300	12,667	11,300	12,667	Manual
2	Unknown	7,633	2539737	70569	26,118	19,581	16,400	17,792	16,400	17,792	Manual

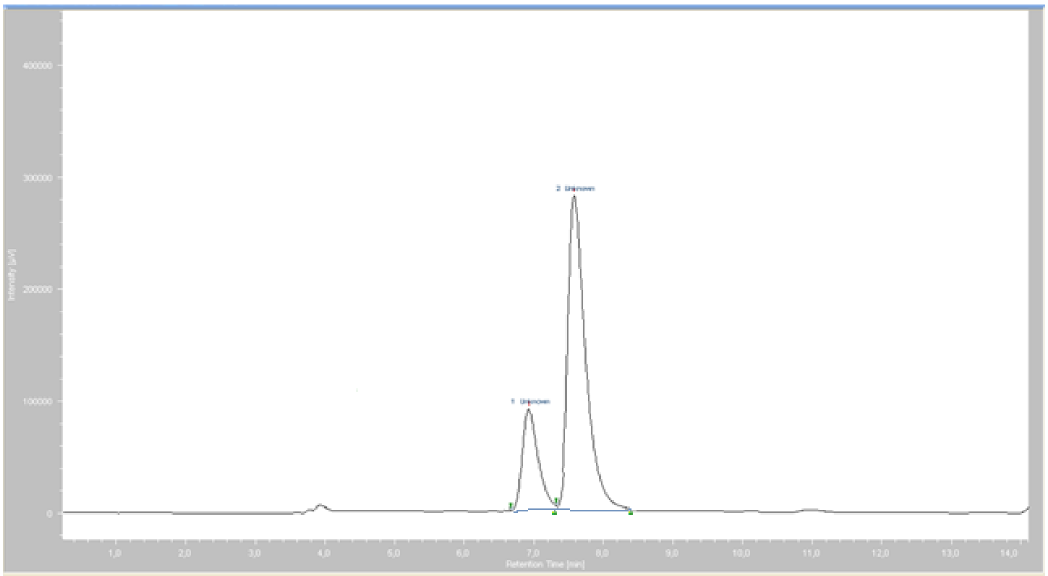

Peak Info. Table

#	Peak Name	tR	Area	Height	Area%	Height%	Peak Start	Peak End	Base Start	Base End	Peak Mark
1	Unknown	7,108	6667948	217244	74,614	81,594	11,467	13,158	11,467	13,158	Manual
2	Unknown	7,676	1904429	49007	25,486	18,406	16,817	18,450	16,817	18,450	Manual

Figure S7. Spectra of substrate **IX** with **C3** (47 % e.e.) and **C4** (48 % e.e.) respectively.

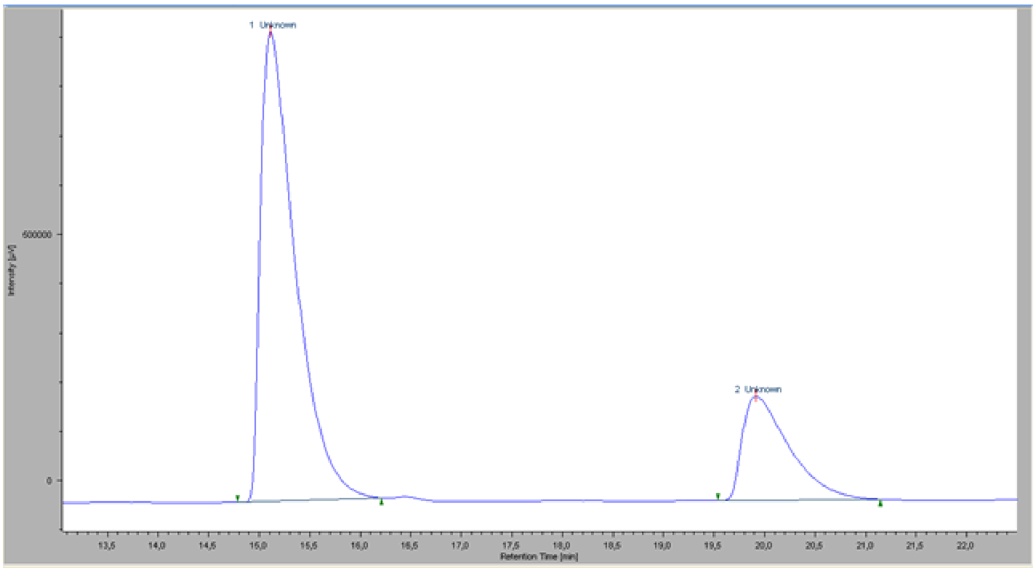

Peak Info. Table

#	Peak Name	tR	Area	Height	Area%	Height%	Peak Start	Peak End	Base Start	Base End	Peak Mark
1	Unknown	15,108	22810450	952349	77,048	81,866	14,783	16,208	14,783	16,208	Manual
2	Unknown	19,908	6794916	210951	22,952	18,134	19,533	21,142	19,533	21,142	Manual

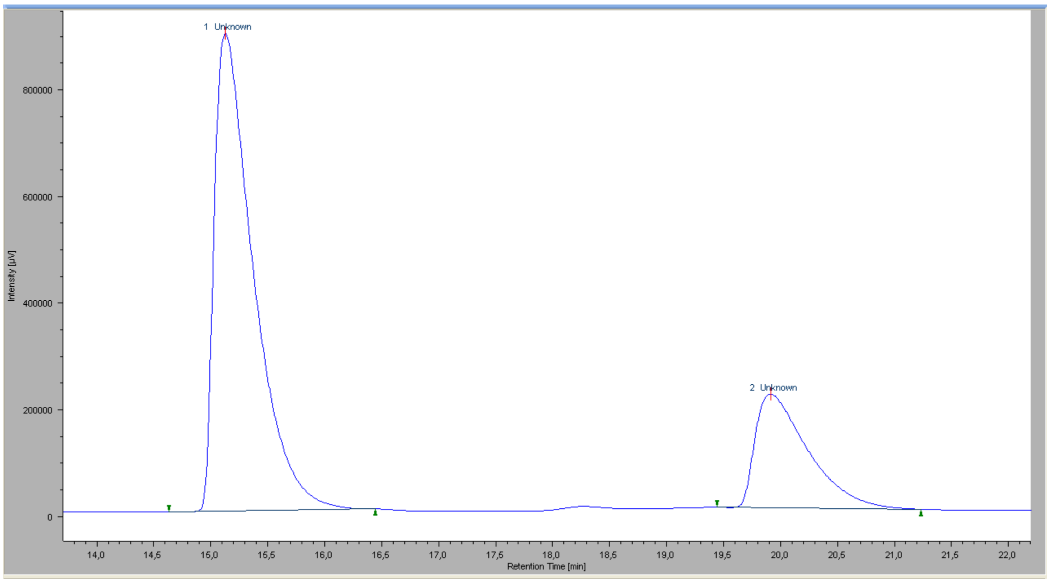

Peak Info. Table

#	Peak Name	tR	Area	Height	Area%	Height%	Peak Start	Peak End	Base Start	Base End	Peak Mark
1	Unknown	15,125	21324528	893795	75,687	80,764	14,633	16,442	14,633	16,442	Manual
2	Unknown	19,908	6850100	212879	24,313	19,236	19,442	21,225	19,442	21,225	Manual

Figure S8. Spectra of substrate **X** with **C3** (55 % e.e.) and **C4** (51 % e.e.) respectively.

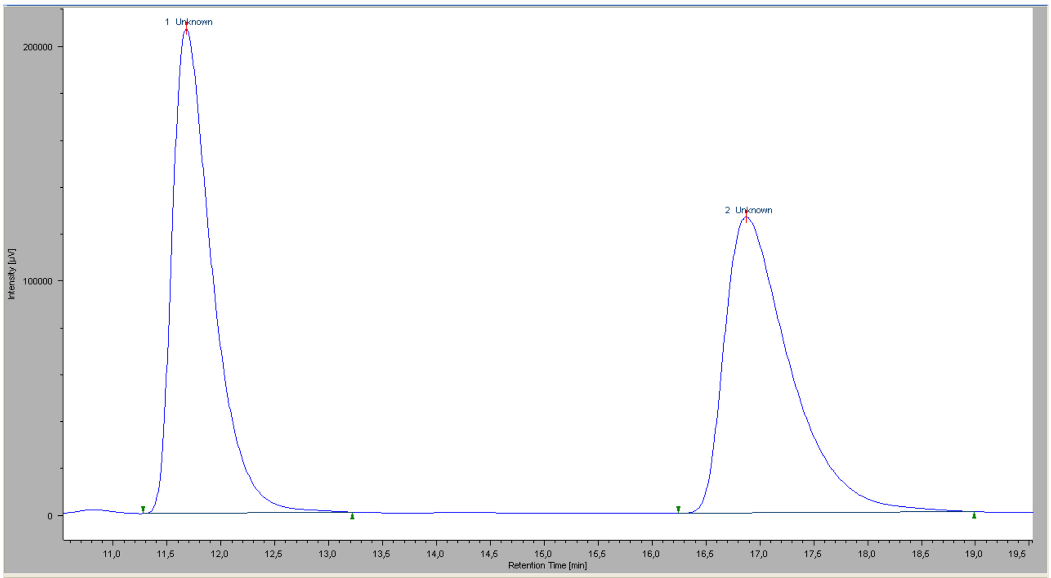

Peak Info. Table

#	Peak Name	tR	Area	Height	Area%	Height%	Peak Start	Peak End	Base Start	Base End	Peak Mark
1	Unknown	11,675	5328877	206497	50,020	62,070	11,275	13,217	11,275	13,217	Manual
2	Unknown	16,867	5324685	126187	49,980	37,930	16,242	18,983	16,242	18,983	Manual

Figure S9. Spectra of substrate **I** with **C8**.
